# Supplementary material for: Characterization of Metabolic Correlations of Ursodeoxycholic Acid with Other Bile Acid Species through In Vitro Sequential Metabolism and Isomer-Focused Identification
Source: Molecules. 2023 Jun 16;28(12):4801. doi: 10.3390/molecules28124801 (PMC10300935; doi:10.3390/molecules28124801)
Supplement: Supplementary file 1 [file molecules-28-04801-s001.zip › molecules-2448643-supplementary.pdf]

Supplemental information

Characterization of Metabolic Correlations of Ursodeoxycholic Acid with Other Bile Acid Species through In Vitro Sequential Metabolism and Isomer-Focused Identification

Wei Li [1], Wei Chen [1], Xiaoya Niu [1], Chen Zhao [2], Pengfei Tu [1], Jun Li [1], Wenjing Liu [3,*] and Yuelin Song [1,*]

1 Modern Research Center for Traditional Chinese Medicine, Beijing Research Institute of Chinese Medicine, Beijing University of Chinese Medicine, Beijing 100029, China; lw160221071@163.com (W.L.)

2 Zhangzhou Pien Tze Huang Pharmaceutical Co., Ltd., Zhangzhou 363000, China

3 School of Pharmacy, Henan University of Chinese Medicine, Zhengzhou 450046, China

* Correspondence: liuwj107@163.com (W.L.); syltwc2005@163.com (Y.S.)

.

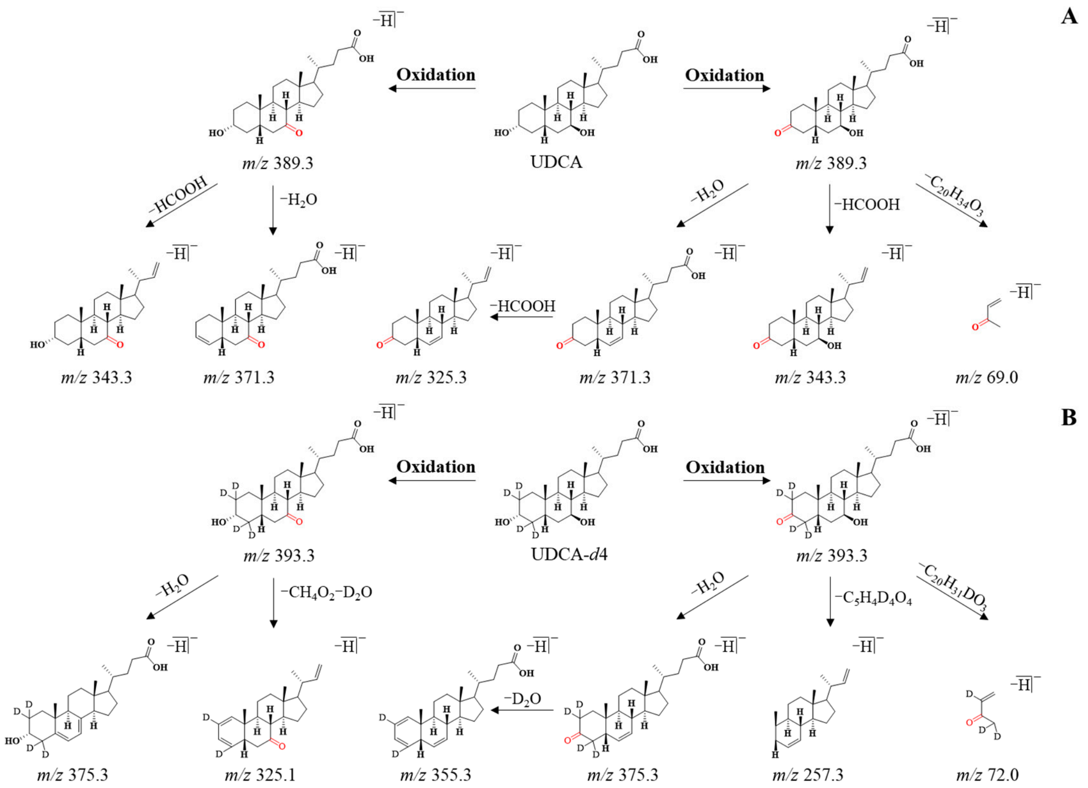

Figure S1. LC–MS chromatogram and proposed fragmentation pathways of oxidative products in the negative ion mode. (A) Proposed fragmentation pathways of 7*β*-hydroxy-3-oxo-5*β*-cholan-24-oic acid and 7-ketolithocholic acid. (B) Proposed fragmentation pathways of 7*β*-hydroxy-3-oxo-5*β*-cholan-24-oic acid- and 7-ketolithocholic acid-d_4.

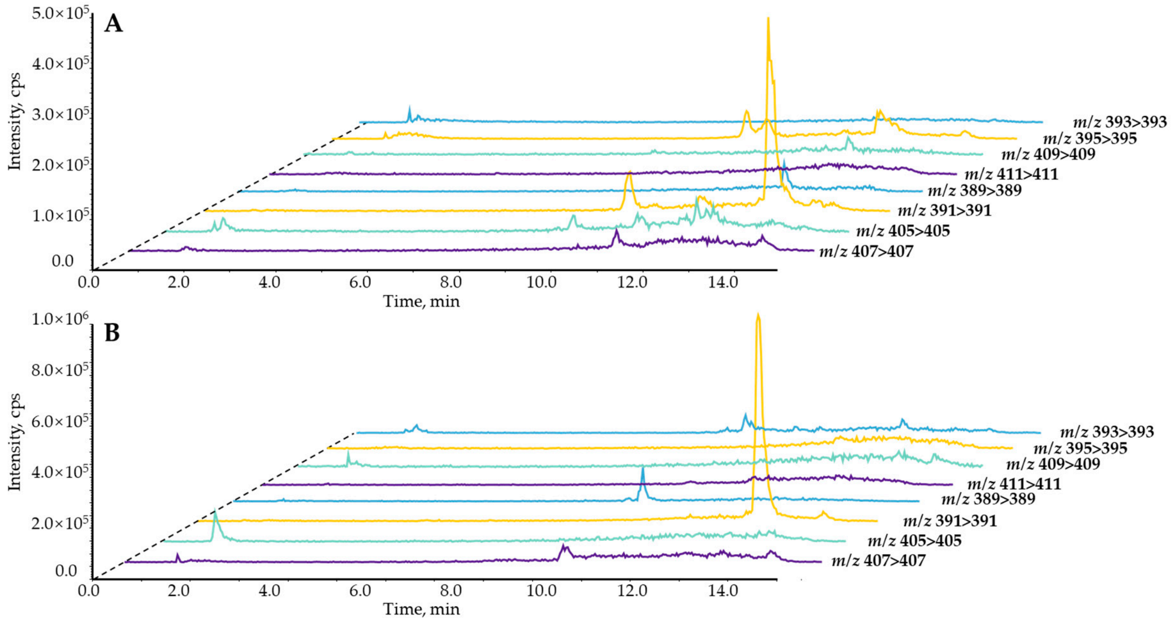

Figure S2. Extraction chromatography of incubation system from the LC–*p*MRM program (A) without UDCA and with NADPH incubation system involved HLM; (B) without UDCA and NADPH incubation system, with HLM.

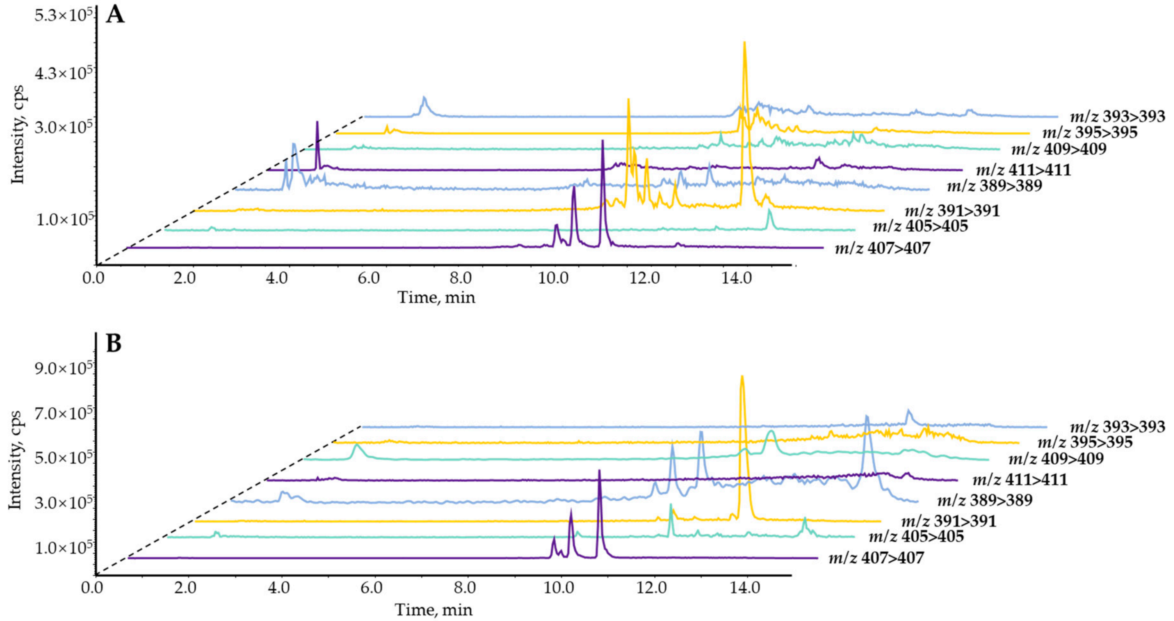

Figure S3. Extraction chromatography of incubation system from the LC–*p*MRM program (A) without UDCA and with NADPH incubation system involved MLM; (B) without UDCA and NADPH incubation system, with MLM.

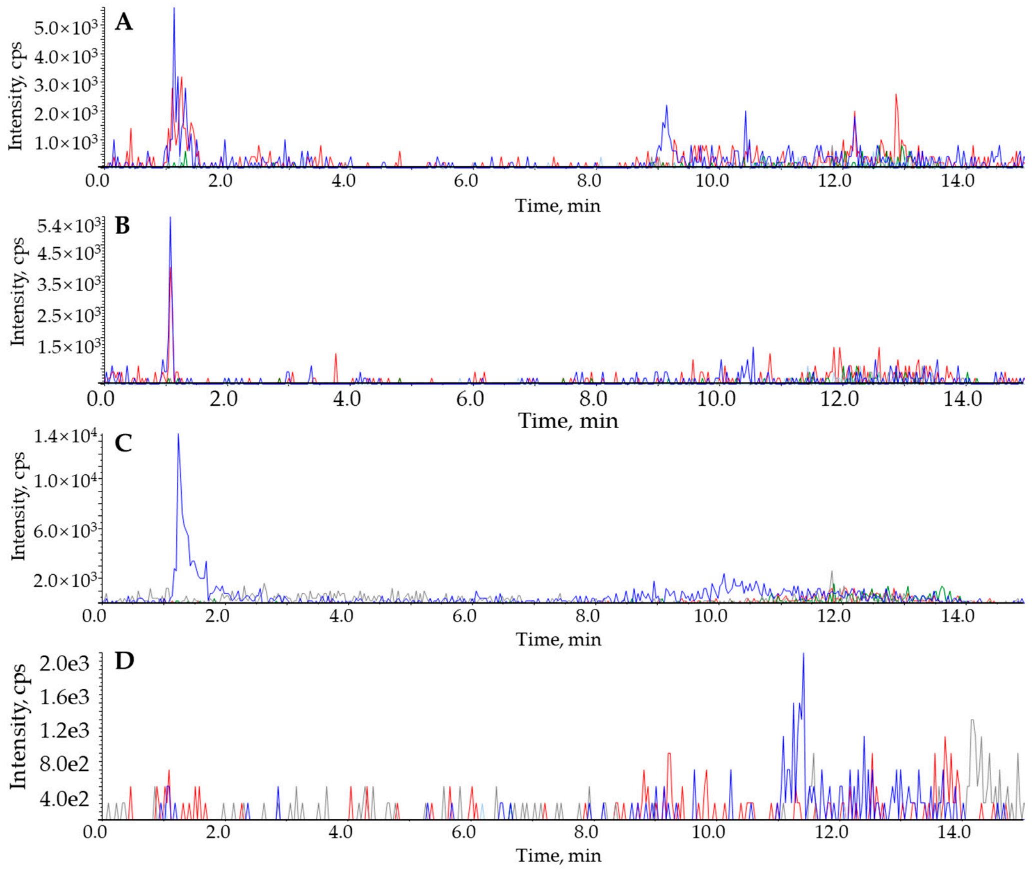

Figure S4. Extraction chromatography of incubation system from the LC–*p*MRM program (A) without UDCA and with UDPGA incubation system involved HLM or MLM; (B) without UDCA and UDPGA incubation system, with HLM or MLM; (C) without UDCA and with PAPS incubation system involved HLM; (D) without UDCA and PAPS incubation system, with HLM or MLM.

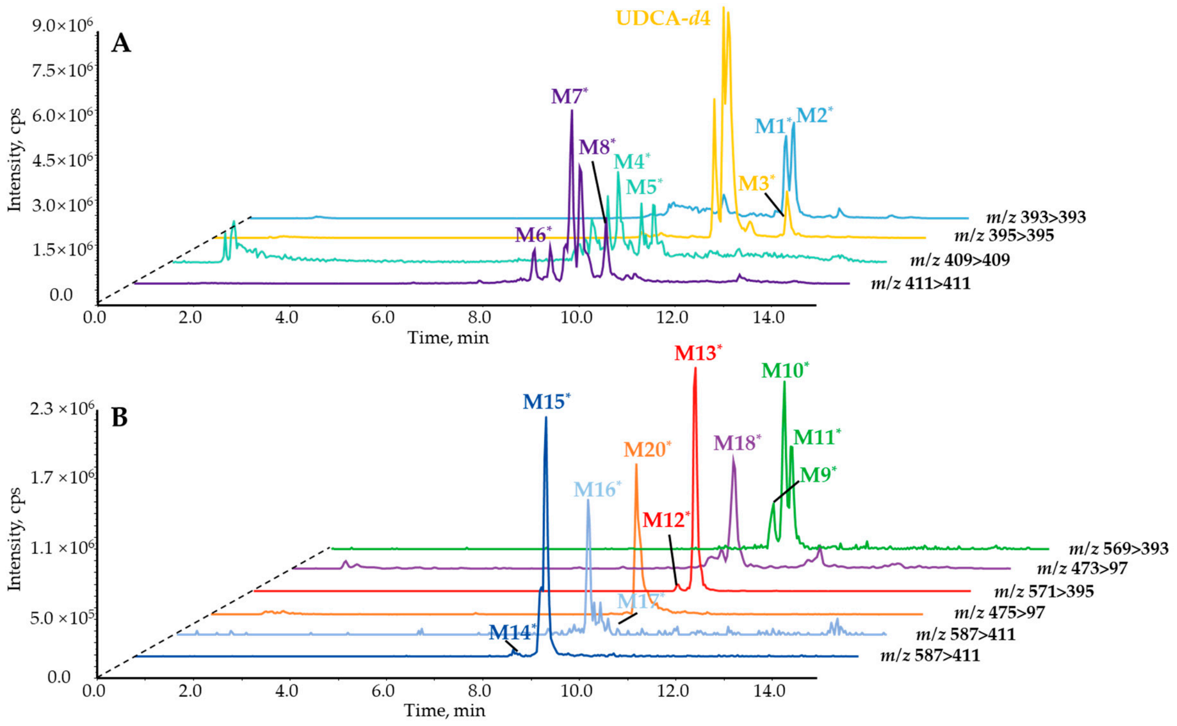

Figure S5. (A) Extracted ion current chromatogram (phase I metabolites of UDCA-d_4) from the LC−*p*MRM program; (B) Extracted ion current chromatogram (phase II glucuronidated and sulfated metabolites of UDCA-d_4) from the LC−*p*MRM program.

Table S1 The chromatographic, MS/MS information, optimized ion transitions and collision energy information of UDCA *in vitro* metabolites generated from incubation of liver microsomes (human and mouse) by *p*MRM program.

Metabolite	t_R (min)	Q1 (Da)	Q3 (Da)	OCE (eV)	DP (V)	MS^1 (Da)	MS^2 (Da)	AF2 level corresponding to 50% relative intensity	Compound name	Incubation system
**M1**	10.99	389.3	389.3	–22.8	–100	–	–	*m/z* 389.3>389.3>389.3(0.01802V)	7*β*-hydroxy-3-oxo-5*β*-cholan-24-oic acid	HLM & MLM
	8.66	–	–	–	–	389.2705	371.2637,343.2656,325.2553,69.0346			MLM
**M1-*d*$_4$**	8.63	393.3	393.3	–22.8	–100	393.2952	375.2863,355.2267,257.1544,72.0535	–	7*β*-hydroxy-3-oxo-5*β*-cholan-24-oic acid-*d*4	HLM & MLM
**M2**	11.25	389.3	389.3	–22.3	–100	–	–	*m/z* 389.3>389.3>389.3(0.02379V)	7-ketolithocholic acid*	HLM & MLM
	8.78	–	–	–	–	389.2705	371.2610,343.2649			MLM
**M2-*d*$_4$**	8.79	393.3	393.3	–22.3	–100	393.2954	375.2910,325.1848	–	7-ketolithocholic acid-*d*4	HLM & MLM
**M3**	12.00	391.3	391.3	–20.1	–100	–	–	*m/z* 391.3>391.3>391.3(0.02077V)	Chenodeoxycholic acid*	HLM & MLM
	8.34	–	–	–	–	391.286	373.2764,345.7297			
**M3-*d*$_4$**	9.53	395.3	395.3	–20.1	–100	395.3117	377.3023	–	Chenodeoxycholic acid-*d*4	HLM & MLM
**M4**	9.40	405.3	405.3	–16.79	–100	–	–	–	3*α*,7*β*-dihydroxy-6-oxo-5*β*-cholan-24-oic acid or 3*α*,6*β*-Dihydroxy-7-oxo-5*β*-cholan-24-oic acid	HLM & MLM
	7.69	–	–	–	–	405.2659	387.2552,375.2551,369.2458,357.2482			HLM
**M5**	10.12	405.3	405.3	–20.67	–100	–	–	–	3*α*,7*β*-dihydroxy-12-oxo-5*β*-cholan-24-oic acid	HLM & MLM
	7.98	–	–	–	–	405.2659	387.2559,353.2128,69.0345			MLM
**M5-*d*$_4$**	7.94	409.3	409.3	–20.67	–100	409.2907	391.2817	–	3*α*,7*α*-dihydroxy-12-oxo-5*β*-cholan-24-oic acid -*d*$_4$	MLM
**M6**	8.30	407.3	407.3	–21.02	–100	–	–	*m/z* 407.3>407.3>407.3(0.02594V)	3*β*,7*β*,12*α*-trihydroxy-5*β*-	HLM

	6.60	–	–	–	–	407.2815	389.2744,371.2604		cholan-24-oic acid	
**M6-*d_4***	6.58	411.3	411.3	–21.02	–100	411.3063	375.2857,365.3013,69.0346	–	3*β*,7*β*,12*α*-trihydroxy-5*β*-cholan-24-oic acid-*d^4*	HLM
**M7**	9.11	407.3	407.3	–18.1	–100	–	–	*m/z* 407.3>407.3>407.3(0.03270V)	Ursocholic acid *	HLM & MLM
	6.70	–	–	–	–	407.2813	391.2476,345.2444,271.2069			HLM
**M7-*d_4***	6.70	411.3	411.3	–18.1	–100	411.3065	395.2760,349.2695	–	Ursocholic acid-d_4	MLM
**M8**	9.86	407.3	407.3	–22.3	–100	–	–	*m/z* 407.3>407.3>407.3(0.03836V)	*β*-muricholic acid *	HLM & MLM
	7.40	–	–	–	–	407.2812	371.2602,331.2287,69.0347			MLM
**M8-*d_4***	7.37	411.3	411.3	–22.3	–100	411.3063	393.2951,365.1661,349.2690	–	*β*-Muricholic acid-*d_4*	MLM
**M9**	9.16	565.3	389.3	–47.68	–100	–	–	*m/z* 565.3>389.3>389.3(0.02630V)	7*β*-hydroxy-3-oxo-5*β*-cholan-24-oic acid-7-*O*-glucuronide	HLM & MLM
**M10**	9.37	565.3	389.3	–48.38	–100	–	–	*m/z* 565.3>389.3>389.3(0.02491V)	7*β*-hydroxy-3-oxo-5*β*-cholan-24-oic acid-24-*O*-glucuronide	HLM & MLM
**M11**	9.52	565.3	389.3	–49.74	–100	–	–	*m/z* 565.3>389.3>389.3(0.01962V)	7-ketolithocholic acid-24-*O*-glucuronide	HLM & MLM
**M12**	8.83	567.3	391.3	–49.96	–100	–	–	*m/z* 567.3>391.3>391.3(0.02576V)	Ursodeoxycholic acid-7-*O*-glucuronide	HLM & MLM
**M13**	9.16	567.3	391.3	–51.5	–100	–	–	*m/z* 567.3>391.3>391.3(0.03289V)	Ursodeoxycholic acid-3-*O*-glucuronide	HLM & MLM
	6.65	–	–	–	–	567.3202	391.2863,175.0256,129.0200,113.0250			MLM
**M13-*d_4***	6.62	571.3	395.3	–51.5	–100	571.345	395.3119,175.0255,133.0149,113.0250	–	Ursodeoxycholic acid-3-*O*-glucuronide-*d_4*	MLM
**M14**	7.78	583.3	407.3	–50.57	–100	–	–	*m/z* 583.3>407.3>407.3(0.03090V)	Ursocholic acid-7-*O*-	MLM

									glucuronide	
**M15**	8.44	583.3	407.3	–51.33	–100	–	–	*m/z* 583.3>407.3>407.3(0.03688V)	*β*-Muricholic acid-3-*O*-glucuronide	MLM
	6.00	583.3	407.3	–51.33	–100	583.3144	407.2813,175.0255,129.0201,113.0250			
**M16**	8.62	583.3	407.3	–53.74	–100	–	–	*m/z* 583.3>407.3>407.3(0.03289V)	Ursocholic acid-3-*O*-glucuronide	HLM
**M17**	9.2	583.3	407.3	–54.11	–100	–	–	*m/z* 583.3>407.3>407.3(0.02803V)	3*β*,7*β*,12*α*-trihydroxy-5*β*-cholan-24-oic acid-3-*O*-glucuronide	HLM
**M18**	9.22	469.3	97	–99.87	–100	–	–	*m/z* 469.3>389.3>389.3(0.02525V)	7*β*-hydroxy-3-oxo-5*β*-cholan-24-oic acid-7-sulfate	HLM
**M19**	8.69	471.3	97	–102.5	–100	–	–	*m/z* 471.3>391.3>391.3(0.02812V)	Ursodeoxycholic acid-3-sulfate*	HLM & MLM
	6.64	–	–	–	–	471.2427	391.2880,96.9605,79.9576			HLM
**M19-**d_4	6.62	475.3	97	–102.5	–100	475.2677	395.3124	–	Ursodeoxycholic acid-d_4-3-sulfate	HLM & MLM
**M20**	8.92	471.3	97	–101.1	–100	–	–	*m/z* 471.3>389.3>389.3(0.02516V)	Ursodeoxycholic acid-7-sulfate	HLM & MLM

Note：“*” identified by authentic reference. HLM: human liver microsomes; MLM: mouse liver microsomes.
